# Effectiveness of a Parent-Based eHealth Intervention on Physical Activity, Dietary Behaviors, and Sleep in Preschoolers: Randomized Controlled Trial

**DOI:** 10.2196/70886

**Published:** 2025-09-02

**Authors:** Zhou Peng, Li Ming Wen, Patrick W C Lau

**Affiliations:** 1 Department of Sports and Health Sciences Academy of Wellness and Human Development Hong Kong Baptist University Hong Kong China (Hong Kong); 2 School of Public Health, Faculty of Medicine and Health University of Sydney Sydney Australia; 3 Laboratory of Exercise Science and Health Beijing Normal-Hong Kong Baptist University Zhuhai China (Hong Kong)

**Keywords:** physical activity, dietary behaviors, sleep, screen time, energy balance–related behaviors

## Abstract

**Background:**

The prevalence of physical inactivity, unhealthy diet, and sleep disturbance among preschoolers is increasing dramatically. Parents play a crucial role in fostering their children’s physical activity (PA), dietary behaviors, and sleep habits. Face-to-face interventions have barriers such as time commitment, making eHealth options appealing. However, current parent-based eHealth interventions have limitations in design (eg, focus on a single outcome, imbalanced content, and sequence), and their results cannot be generalized to other regions.

**Objective:**

This study aims to assess the effectiveness of parent-based eHealth interventions targeting PA, dietary behaviors, and sleep in preschoolers in China.

**Methods:**

This single-blinded randomized controlled trial with 2 parallel arms comprised a 12-week intervention followed by a 12-week follow-up, conducted from September 2023 to June 2024 in China. The intervention, grounded in social cognitive theory, included 12 interactive modules on PA (n=4), dietary behaviors (n=4), and sleep (n=4), each delivered weekly via social media. Each module consisted of videos, evidence-based information, parent interaction, goal setting, and feedback. The control group received a weekly electronic pamphlet via WeChat without interactive components. Preschoolers’ PA, sleep duration, and sleep quality were assessed using the wGT3X-BT ActiGraph, while dietary behaviors, sleep problems, and screen time were reported by parents through paper-based questionnaires. Generalized estimating equations, adjusting for demographic covariates, were used to examine the effects of the parent-based eHealth intervention on preschoolers’ outcomes.

**Results:**

A total of 237 eligible parent-child dyads were randomly assigned to the intervention group (n=120, mean age 4.51 years, SD 0.72 years; n=67, 55.8%, boys) or the control group (n=117, mean age 4.31 years, SD 0.70 years; n=69, 59%, boys). At baseline, 237 parents completed questionnaires, and 196 preschoolers provided valid ActiGraph data. At posttest, 181 parents completed questionnaires, and 166 preschoolers provided valid ActiGraph data (intervention, n=90; control, n=91). At follow-up, 181 parents again completed questionnaires, and 170 preschoolers provided valid ActiGraph data (intervention, n=90; control, n=91). Educating parents about healthy lifestyles through social media had a positive impact on preschoolers’ light PA (vs control: adjusted mean difference –151.61 minutes/7 days, 95% CI –418.18 to –96.67, *P*=.002, Cohen d=0.28), vigorous PA (vs control: adjusted mean difference 138.47 minutes/7 days, 95% CI 117.61-183.10, *P*=.03, Cohen d=0.23), sleep latency (vs control: adjusted mean difference –21.04 minutes/day, 95% CI –16.07 to –6.00, *P*=.005, Cohen d=0.78), sleep efficiency (vs control: adjusted mean difference 4.61%, 95% CI 4.29-9.72, *P*<.001, Cohen d=0.34), and screen time (vs control: on weekdays, adjusted mean difference –16.42 minutes/weekday, 95% CI –30.83 to –2.01, *P*=.01, Cohen d=0.25; on weekends, adjusted mean difference –73.88 minutes/weekend day, 95% CI –98.48 to –49.28, *P*<.001, Cohen d=.46).

**Conclusions:**

The findings may help address unhealthy lifestyles commonly observed in young children. Further efforts are needed to leverage cutting-edge technological advancements to support families in creating healthy living environments for children across broader regions.

**Trial Registration:**

ClinicalTrials.gov NCT06025019; https://clinicaltrials.gov/ct2/show/NCT06025019

**International Registered Report Identifier (IRRID):**

RR2-10.2196/58344

## Introduction

### Background

Physical activity (PA), dietary behaviors, and sleep are collectively termed “the big three pillars of an individual’s health” [1]. Regular engagement in PA, healthy eating habits, and high-quality sleep are critical for enhancing brain growth and the central nervous system [2,3], promoting cognitive performance [4], improving musculoskeletal health [5], reducing symptoms of mental disorders (eg, anxiety and depression) [6], and lowering the likelihood of developing obesity and noncommunicable diseases (eg, cardiovascular diseases) [7]. Nonetheless, people’s lifestyles have become increasingly characterized by physical inactivity and sedentary behaviors [6], unhealthy dietary habits [8], and disrupted sleep routines [9], all of which can adversely affect lifelong health trajectories [10]. This underscores the importance of establishing healthy lifestyle behaviors from an early age (ie, the preschool years), when behavior patterns are still developing and more likely to persist throughout the lifespan [11].

Given the high attendance rate of preschoolers (approximately 50%-90%) in Early Childhood Education and Care (eg, long day care, nurseries, kindergartens, and family day care), along with the substantial amount of time most children spend in these settings (on average 25-35 hours/week), they represent an ideal context for delivering healthy lifestyle strategies for preschoolers [12]. A recent study indicated that a school-based intervention effectively increased preschoolers’ moderate-to-vigorous physical activity (MVPA) and total PA, while decreasing sedentary time at the 8-week posttest [13]. The results of a systematic review evaluating the effectiveness of childcare-based interventions on preschoolers’ healthy eating showed that most of the reviewed studies (8 out of 14) reported significant improvements, including increased intake of fruits and vegetables and decreased total fat consumption [14]. A meta-analysis further reported that school-based interventions targeting preschoolers’ 24-hour movement behaviors reduced sedentary behavior outcomes and increased sleep duration [15]. Arguably, healthy lifestyle interventions implemented in Early Childhood Education and Care settings could be an effective approach to promoting preschoolers’ healthy lifestyles.

However, recent evidence has highlighted the weaknesses of interventions carried out in Early Childhood Education and Care settings. First, kindergartens demonstrated a lack of fidelity in intervention delivery and faced difficulties in implementation due to high staff turnover rates [16]. In addition, teachers were reluctant to participate, as the intervention diverted attention from other educational activities [17]. Finally, differences in individual teaching styles and interpretations of the intervention led to inconsistent adherence to intervention protocols [18].

In response, extensive studies have highlighted that parental involvement in interventions may be highly valuable for shaping preschoolers’ PA, dietary behaviors, and sleep [19]. In the early years of life, preschoolers are still developing autonomy and rely on parental supervision [20], while parents strongly influence children’s PA, dietary behaviors, and sleep through their regulations and recommendations [21]. However, due to the challenges associated with face-to-face parent-based interventions (eg, time consumption and travel costs) [22], eHealth interventions—defined as the use of the internet and mobile technologies to deliver health-related information [23]—offer appealing alternatives because of their accessibility and lower costs [24].

A previous systematic review highlighted that existing literature examining the impact of parent-based eHealth interventions on preschoolers’ PA, dietary behaviors, and sleep either focused on a single variable among these behaviors or did not balance the intervention content modules (ie, diet-related modules were more extensive than those related to PA and sleep) [25]. Additionally, the sequence in which modules were delivered was often overlooked. Familiarity with later-distributed modules at the posttest may have led to more favorable outcomes for these modules compared with earlier ones [25]. The systematic review further indicated that empirical relationships identified in one culture may stem from its specific context and may not be transferable to other countries [25]. Considerable uncertainty still exists regarding the effectiveness of parent-based eHealth interventions on preschoolers’ PA, dietary behaviors, and sleep in China. Therefore, high-quality, robustly designed parent-based eHealth interventions with balanced modules and well-structured content delivery are needed to determine their effectiveness on Chinese preschoolers’ PA, dietary behaviors, and sleep.

### Objectives

This study aimed to investigate the effectiveness of a parent-based eHealth intervention on PA, dietary behaviors, and sleep among preschoolers raised in a Chinese cultural context.

## Methods

### Study Design

The protocol for this study has been published [26]. This was a single-blinded randomized controlled trial with 2 parallel arms, consisting of a 12-week intervention followed by a 12-week follow-up. It was designed to promote preschoolers’ PA, dietary behaviors, and sleep by providing parents with relevant health information and recommendations through an eHealth modality (ie, videos and WeChat [Tencent Holdings Limited], which is similar to WhatsApp) and by motivating parents to create a family environment with healthy regulations in which their children live. Data were collected at baseline, 12 weeks postbaseline, and 24 weeks postbaseline in 4 kindergartens in Guiyang, Guizhou Province, Mainland China, with assistance from Sun Yat-sen University and Guiyang Preschool Education College, between October 2023 and July 2024. Multimedia Appendix 1 presents the CONSORT (Consolidated Standards of Reporting Trials) checklist.

### Recruitment of Participants and Eligibility Criteria

The entire recruitment process was conducted within the school setting. Kindergarten teachers sent messages to parents in their respective WeChat class groups, providing a brief introduction to the study’s objectives and significance. Parents were then asked to respond with either “yes” or “no” to indicate their willingness to participate. Face-to-face presentations outlining the objectives and procedures of the intervention were conducted in the 4 kindergartens for parents who agreed to participate (ie, those who replied “yes” in the WeChat group). The inclusion criteria were as follows: (1) parents were over 21 years old and had children aged 3-6 years; (2) parental commitment to participate in the full 24-week intervention; (3) access to mobile technology; (4) parents and children were required to be healthy (defined as a state of physical, mental, social, intellectual, and emotional well-being, and the absence of disease) and able to engage in normal PA; and (5) parents had to reside with the participating child for at least 4 days/week.

Children were excluded from the study if they (1) had a diagnosed neurological or physical disability; (2) had a parent with a serious physical or psychological condition that would make participation too demanding for the family; or (3) had special dietary requirements or allergies that would necessitate tailoring the intervention or could be adversely affected by it.

### Ethical Considerations

This intervention study was approved by the Research Ethics Committee of Hong Kong Baptist University (approval number SOSC-SPEH-2022-23_115) and registered in the ClinicalTrials.gov Protocol Registration and Results System under identifier NCT06025019. All preschoolers and their parents were fully informed of the potential risks of the experiment. Eligible participants were asked to add the researchers on WeChat and to sign the informed consent form at the end of the parent meetings. The consent form outlined the points detailed in Textbox 1.

**Outline of the consent form points.**

1. Secondary analysis

- The study permitted secondary analysis without requiring additional consent.

2. Confidentiality

- All personal data collected were anonymized and accessible only to the researchers for research purposes.
- The data would be destroyed immediately after publication of the research report to ensure confidentiality.

3. Compensation

- The study lasted 24 weeks and included 3 testing sessions.
- At the end of each session, parents received a detailed report of their child’s results, providing insight into their child’s PA, dietary behaviors, and sleep.

### Randomization and Blinding

After recruitment, eligible participants were randomized into either the eHealth intervention group or the control group at a 1:1 ratio, with allocation concealment ensured using a computer-generated sequence (SPSS version 27; IBM Corp). The entire allocation process was overseen by the statistician. To maintain strict adherence to the study protocol, only the 2 researchers (ZP and PWCL) responsible for implementing the intervention were informed of group allocation, while the participants remained blinded.

### Sample Size

A priori analysis was conducted using G*Power 3.1.9.6, with an *F*-test ANOVA repeated-measures, within-between interaction design [27]. A recent systematic review and meta-analysis reported a small yet statistically significant effect size for interventions aimed at enhancing PA in children under 5 years old [28]. Additionally, intervention studies targeting improvements in diet and sleep quality in young children have shown small to medium effect sizes [29,30]. Therefore, a small effect size was adopted in this study. A minimum sample size of 82 parent-child dyads/group (intervention and control) was required to detect a small effect size (Cohen *d*=0.3) with a power of 0.80 and an α level of .05. Considering a potential 20% attrition rate [31], a total of 206 parent-child dyads were needed (103 in each group).

### The Rationale of the Intervention

The intervention was implemented in accordance with the previously published protocol [26]. Briefly, it was grounded in social cognitive theory, which posits that learning is a social phenomenon in which individuals acquire knowledge by observing, imitating, and modeling the behaviors of others [32]. For preschool-age children, observing and mimicking parental behaviors is a fundamental way of learning, as they absorb information from the family environment to develop social, cognitive, and emotional skills [20]. Therefore, a key component of this intervention was to motivate parents to model healthy PA, dietary behaviors, and sleep routines, while fostering a home environment with supportive regulations in these areas.

Social cognitive theory proposes that an individual’s behavior can be modified through the interaction of personal, environmental, and behavioral factors [33]. Personal factors refer to an individual’s self-efficacy in carrying out a behavior, shaped by personality, knowledge, beliefs, self-perceptions, and expectations. Environmental factors are defined as supportive contexts that facilitate the performance of a behavior. Behavioral factors refer to an individual’s response once a behavior has been performed. Together, these 3 factors outline the key intervention components needed to induce behavioral change.

Additionally, social cognitive theory outlines 4 processes for behavior change: attention, retention, reproduction, and motivation [34]. If any of these processes are absent, learning and adoption of new behaviors will not occur [34]. These 4 processes inform the sequence that interventions should follow to induce behavioral change.

### Parent-Based eHealth Intervention

The eHealth intervention comprised 12 interactive modules focused on preschoolers’ PA, dietary behaviors, and sleep, with each theme consisting of 4 modules. These 12 modules were rotated weekly with a theme over the 12-week period. Participants were organized into 26 WeChat groups, each containing 8 members, based on the optimal group size for interaction [35]. Trained facilitators—kindergarten teachers with expertise in PA, diet, and sleep—were recruited for each group and blinded to the study objectives. Their responsibilities included sending videos, facilitating interactions, answering questions, helping parents set goals for their children, and providing feedback, as well as revising goals. Parents received 2 self-monitoring SMS text messages/week: the first, sent midweek, requested feedback on goal progress. A response of “N” triggered a skill-building suggestion, while a response of “Y” received positive reinforcement. The second message, sent on weekends, assessed goal attainment before introducing the new weekly theme.

Based on the sequence outlined in social cognitive theory, participants received the following content each week: (1) Attention—matched with personal factors—emphasizes that individuals cannot learn unless they pay attention to what is happening around them [36]. To address this, attention was promoted by providing printed educational materials and videos through WeChat [37]. At the beginning of each week, parents received a pre-prepared poster and a 3-minute video related to the weekly theme. These videos, recorded by the researchers and structured around behavior change techniques, included preschoolers’ lifestyle recommendations from the World Health Organization, benefits of the target behaviors, common barriers, goal setting, and feedback. (2) Retention—aligned with environmental factors—emphasizes that individuals must not only recognize observed behaviors but also remember them for future application [36]. Therefore, retention was addressed through interactions with other participants in WeChat groups, which helped parents recall the content of the videos and educational materials. Facilitators encouraged communication within these groups, where parents shared their children’s activities, meal photos, and strategies for reducing sedentary behavior. The groups were monitored by the researchers to ensure that discussions aligned with evidence-based guidelines. Parents who did not participate in group exchanges received private reminder messages from the facilitator via WeChat. (3) Reproduction, which is matched with behavioral factors, refers to the individual’s ability to be physically and mentally capable of performing the observed behaviors [36]. Reproduction was supported through goal setting, action planning, and problem-solving around barriers. Facilitators worked with parents privately to establish individualized goals related to PA, dietary behaviors, and sleep for their children, and to refine these goals using the SMART (Specific, Measurable, Achievable, Relevant, and Time-bound) framework. Parents also had regular opportunities to interact with facilitators to address challenges and adjust their plans as needed. (4) Motivation, which is matched with behavioral factors, refers to the individual’s interests and sense of achievement in relation to the tasks and activities being implemented [36]. Motivation was fostered by creating cognitive dissonance, prompting parents to reflect on their children’s current behaviors and to identify the positive outcomes and expectations associated with adopting the planned behaviors. Facilitators also provided 2 types of individualized feedback—based on both group interactions and progress toward goal achievement. First, graphical visualizations of reported PA, diet, and sleep were shared to motivate compliance with World Health Organization recommendations. Second, a “traffic light” poster was used to indicate compliance (green: met; yellow: close; and red: not met). For goals not met, facilitators provided tailored solutions, tips, and reinforcement strategies privately via WeChat.

### Control Group

#### Overview

Parent-child dyads in this group received electronic materials related to the weekly theme at the beginning of each week via WeChat, but did not have access to interactive components.

#### Measurements

Demographic information was collected at baseline. Children’s PA, dietary behaviors, and sleep were assessed at 3 time points using the same procedures: baseline (October 2023) after participant recruitment, posttest (January–February 2024) at the end of the intervention, and follow-up (June 2024), conducted 12 weeks after the intervention was completed. Before data collection, researchers grouped children according to the number of available ActiGraph wGT3X-BT accelerometers and informed parents that the devices should be worn for 7 consecutive days. If a child was expected to be absent during the collection period, group adjustments were made. On the scheduled day, researchers fitted the first group of children with accelerometers in the kindergarten and instructed teachers to monitor their use during school hours, lunch, and after school. When parents picked up their children wearing accelerometers, they were invited to spend approximately 20 minutes in a separate room at the kindergarten to complete paper-based questionnaires on preschoolers’ dietary behaviors, sleep problems, and screen time. Completed questionnaires were returned to the teachers. After 1 week of data collection for each group, researchers retrieved the ActiGraph wGT3X-BT accelerometers, downloaded and initialized the data, recharged the devices, and repeated the same procedure with the subsequent groups. All questionnaire items and parent-reported responses were manually entered into a Microsoft Excel sheet by a research assistant for further analysis. A second research assistant then conducted a double check to ensure accuracy. Both assistants were blinded to the research objectives and group allocation.

#### Demographic Information

Parents’ information included socioeconomic status (income range, educational level, and occupational status), age, gender, marital status, and number of children. Children’s information included gender, age, height, and weight. Height and weight were measured using the SECA Medical Body Composition Analyzer, with weight recorded to the nearest 0.1 kg (children barefoot and in light clothing) and height to the nearest 0.1 cm. Parents’ BMI was calculated from self-reported height and weight (kg/m^2^) and categorized using cutoff points for Asian adults (18.5-22.9 kg/m^2^=normal weight; ≥23 kg/m^2^=overweight/obesity) [38]. Preschoolers’ BMI was calculated from measured height and weight (kg/m^2^). The BMI score was then categorized as normal weight or overweight based on the age- and gender-specific standards provided by the International Cutoff Points for Identification of Overweight and Obesity in Children (kg/m^2^) [39].

#### Preschoolers’ PA

The PA level and sedentary time were objectively monitored using a triaxial accelerometer (ActiGraph GT3X-BT). The ActiGraph accelerometer is a valid and reliable tool for objectively measuring PA levels in preschoolers [40]. Kindergarten teachers and parents received written and video instructions on its use. In addition, parents were asked to keep an activity diary for both wear and nonwear time. Teachers checked accelerometer wear on each school day. An accelerometer was affixed to the children’s right wrist to monitor all activities over 7 consecutive days, except during water-related activities or situations that could damage the device, such as swimming or bathing. The ActiLife software (version 6.13) was used to initialize the device and analyze the data. A recording epoch of 1 second and 60 seconds was downloaded for PA and sleep analysis, respectively [40]. Valid wear time was defined as at least 16 hours of wear on a minimum of 3 days (2 weekdays and 1 weekend day) [40]. Nonwear time was defined as 20 consecutive minutes of 0 counts per minute (CPM), following ActiLife standard procedures. Accelerometers were initialized at a sampling rate of 30 Hz and reintegrated into 60-second epochs for analysis. Activity counts were categorized into intensity levels (ie, sedentary behavior, light physical activity [LPA], moderate physical activity [MPA], and vigorous physical activity [VPA]) using the following cutoff points: sedentary, <819 CPM; LPA, 820-3907 CPM; MPA, 3908-6111 CPM; and VPA, ≥6112 CPM [41]. Additionally, a log sheet was provided for parents to record when the device was removed and the reasons for its removal.

#### Preschoolers’ Dietary Behaviors

Children’s dietary behaviors were assessed using the Children’s Eating Behavior Questionnaire, which has been validated in Chinese preschool-age children [42]. Parents rated the frequency of their child’s eating behaviors on a 5-point scale (1=never, 2=rarely, 3=sometimes, 4=often, 5=always) across 8 domains (35 items): satiety responsiveness (eg, My child has a big appetite), slowness in eating (eg, My child finishes meals quickly), food fussiness (eg, My child refuses new food at first), food responsiveness (eg, My child is always asking for food), enjoyment of food (eg, My child looks forward to mealtimes), desire to drink (eg, My child is always asking for a drink), emotional undereating (eg, My child eats less when angry), and emotional overeating (eg, My child eats more when annoyed). Items marked with an asterisk were reverse scored (5 to 1 instead of 1 to 5). For each subscale, higher mean scores indicated greater expression of the corresponding eating behavior. In addition, parents were given a modified log sheet to record all foods their child consumed during the 5 main meals (breakfast, morning snack, lunch, afternoon snack, and dinner). They were also asked to note the time of any additional snacks consumed. Depending on the category, very high or very low levels indicate unhealthy eating behaviors; however, there are no established cutoff values to classify behaviors as dysfunctional. The scales demonstrated high internal consistency reliability, with an overall Cronbach α above 0.7 [42].

#### Preschoolers’ Sleep Duration and Quality

The ActiGraph accelerometer was used to assess children’s sleep latency and sleep efficiency. Bedtime and wake-up time were determined using the algorithm by Sadeh et al [43], while periods of sleep were estimated with the algorithm by Tudor-Locke et al [44]. Duration of nighttime sleep, bedtime, and wake-up time were analyzed in 60-second epochs using ActiLife software (version 6.13) and cross-checked against parent-completed log sheets. Sleep data were considered valid if the device was worn for at least three nights [43].

#### Preschoolers’ Sleep Problems

Children’s sleep problems were assessed using the Chinese version of the Children’s Sleep Habits Questionnaire [45], a widely used parent-report survey for screening children aged 3-10 years. The questionnaire contains 33 items across 8 domains: bedtime resistance, sleep onset delay, sleep duration, sleep anxiety, night waking, parasomnia, sleep-disordered breathing, and daytime sleepiness. Each item is rated on a 3-point scale: “usually” if the behavior occurs 5 or more times/week (scored 3), “sometimes” if it occurs 2-4 times/week (scored 2), and “rarely” if it occurs once or not at all during the week (scored 1). Higher scores indicate greater sleep problems, with a total score above 41 suggesting a sleep disorder. Items 1, 2, 3, 10, 11, and 26 were reverse scored as follows: 1=3, 2=2, 3=1. The remaining items (4-9, 12-25, 27-32b) retained their original scores. The questionnaire has demonstrated good reliability and validity, with a Cronbach α of 0.73 [45].

#### Preschooler’s Screen Time

Parents were asked to answer questions estimating their children’s usual screen time on a typical weekday and weekend to calculate the average weekly screen time. The questionnaire also assessed the availability of screens and rules regarding screen use. This 4-item questionnaire has been used in previous studies and was translated into Chinese using a back-to-back translation method [46]. For example, “Do you have rules regarding screen time at home?” (A=yes, 1 point; B=no, 2 points). “Does your child’s bedroom have any screen devices?” (A=yes, 1 point; B=no, 2 points). “How often does your child watch TV while eating?” (A=never, 1 point; B=1-3 times/week, 2 points; C=4-6 times/week, 3 points; and D=7+ times/week, 4 points). “On average, how much time does your child spend in front of a screen on weekdays (Monday to Friday) and on weekends each day?”

### Statistical Analysis

Baseline characteristics of the intervention and control groups were compared using the chi-square test for categorical variables (preschoolers’ gender, number of children with overweight and obesity, and parents’ gender, educational level, income range, marital status, number of children in the household, and number of parents with overweight and obesity) and independent *t* tests for continuous variables (preschoolers’ and parents’ age, height, weight, and BMI). An intention-to-treat analysis was conducted. Data from the wGT3X-BT ActiGraph (ie, preschoolers’ PA, sleep quality, and sleep duration) and parent-reported questionnaires (ie, preschoolers’ dietary behaviors, sleep problems, and screen time) were analyzed using generalized estimating equations. Group and time were included as main effects, with demographic variables as covariates (preschoolers’ gender, age, height, weight, BMI; parents’ age, height, weight, gender, educational level, income range, marital status, number of children in the household, and BMI). This analysis examined the effects of the parent-based eHealth intervention on outcomes, including preschoolers’ PA, sedentary behaviors, dietary behaviors, sleep problems, sleep latency, sleep efficiency, sleep duration, and screen time. Pairwise comparisons with Bonferroni adjustment were conducted to examine differences between the intervention and control groups whenever a significant group × time interaction was observed. Missing values were not imputed, as generalized estimating equations provide a regression framework that appropriately handles missing data and does not require the assumption of normally distributed data [47]. The magnitude of the parent-based eHealth intervention on outcomes was expressed as effect size (Cohen *d*), calculated using effect size calculators based on the formula provided by Sullivan and Feinn [48]. Effect sizes were interpreted as small (0.2), medium (0.5), and large (0.8) [48].

## Results

### Characteristics of Participants at Baseline

**Figure 1:**
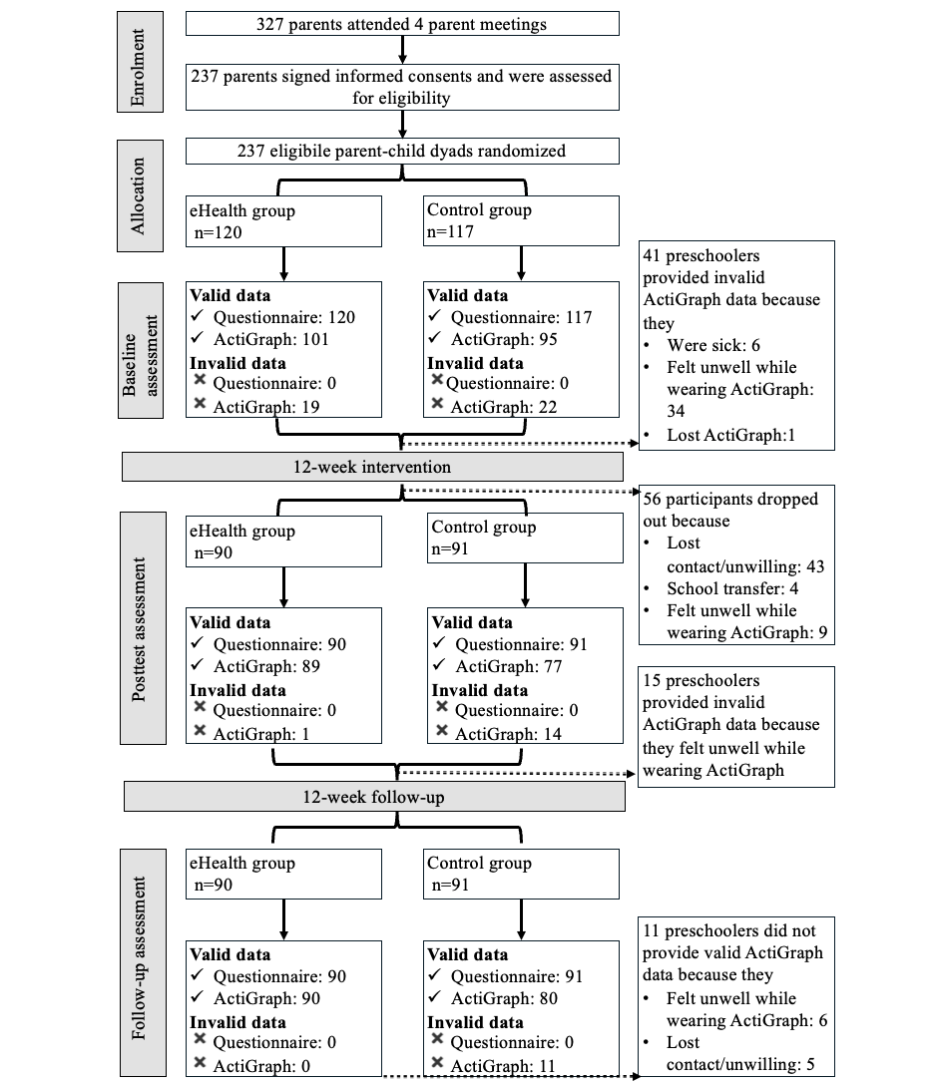
Flowchart of data collection, including reasons for attrition and invalid data.

Figure 1 shows the flow of participants through the study. Baseline data for the parent-child dyads are presented in Table 1. A total of 327 parents attended the 4 face-to-face meetings, of whom 237 were eligible, signed informed consent, and were randomly assigned to the intervention (n=120) or control group (n=117). In the intervention group, the mean age of preschoolers was 4.51 (SD 0.72) years, and 67 (55.8%) children were boys. The mean age of parents in the intervention group was 34.28 (SD 4.74) years, and 93 parents (77.5%) were mothers. In the control group, preschoolers had a mean age of 4.31 (SD 0.70) years, with 69 (59%) children being boys. Parents in the control group had a mean age of 34.88 (SD 3.99) years, and 78 (66.7%) parents were mothers. All demographic characteristics were similar between the 2 groups, except for parents’ height (*P*=.005) and the number of parents with overweight/obesity (*P*=.045).

**Table 1:** Demographic information of participants at baseline between the 2 groups.^a^

Demographic characteristics				Intervention group (n=120)			Control group (n=117)		*P* value^b^
**Preschoolers**									
	Age (years), mean (SD)		4.51 (0.72)			4.31 (0.70)		.75	
	**Gender, n (%)**							.62	
		Boy			67 (55.8)	69 (59.0)			
		Girl			53 (44.2)	48 (41.0)			
	Height (m), mean (SD)		1.09 (0.08)			1.11 (0.07)		.82	
	Weight (kg), mean (SD)		19.56 (4.90)			19.94 (5.84)		.47	
	BMI (kg/m^2^), mean (SD)		16.50 (3.74)			16.19 (4.51)		.46	
	Overweight/obese, n (%)		26 (21.7)			20 (17.1)		.34	
**Parents**									
	Age (years), mean (SD)		34.28 (4.74)			34.88 (3.99)		.67	
	**Gender, n (%)**							.06	
		Mother	93 (77.5)			78 (66.7)			
		Father	27 (22.5)			39 (33.3)			
	Height (m), mean (SD)		1.61 (0.06)			1.63 (0.08)		*.005*	
	Weight (kg), mean (SD)		59.09 (14.39)			64.06 (20.42)		.09	
	BMI (kg/m^2^), mean (SD)		22.59 (5.29)			24.08 (6.58)		.29	
	Overweight/obese, n (%)		44 (36.7)			58 (49.6)		*.04*	
	Only 1 child, n (%)		30 (25.0)			43 (36.8)		.05	
	**Marital status, n (%)**							.72	
		Married	115 (95.8)			111 (94.9)			
		Divorced	5 (4.2)			6 (5.1)			
	**Education, n (%)**							.99	
		College and below	52 (43.3)			51 (43.6)			
		Bachelor	56 (46.7)			56 (47.9)			
		Master and above	12 (10.0)			10 (8.5)			
	**Income (RMB^c^ per month), n (%)**							.10	
		RMB≤3000	4 (3.3)			11 (9.4)			
		3001<RMB≤7345	30 (25.0)			38 (32.5)			
		7346<RMB≤20,000	72 (60.0)			57 (48.7)			
		RMB>20,000	14 (11.7)			11 (9.4)			

^a^Mean (SD) was used to describe continuous variables, whereas n (%) was used for categorical variables.

^b^Italicized *P* values indicate that all the demographic information between the 2 groups was not significantly different except for the parents’ height (*P*=.005) and the number of those with overweight/obesity (*P*=.045).

^c^1 RMB=US $0.14.

### Effectiveness of Parent-Based eHealth Intervention on Preschoolers’ PA

Descriptive information and changes over time are presented in Tables 2 and 3. Differences between the 2 groups are shown in Table 4.

**Table 2:** Summary of descriptive data for each outcome variable (n=237).

Variable				Baseline (week 0), mean (SD)				Posttest (week 12), mean (SD)				Follow-up (week 24), mean (SD)					
				Intervention		Control		Intervention		Control		Intervention		Control			
**Physical activity**																	
	Light physical activity (minutes/7 days)		2064.08 (37.68)		1958.26 (40.56)		1060.70 (33.99)		1212.31 (53.11)		1849.26 (40.77)		1851.23 (39.67)				
	Moderate physical activity (minutes/7 days)		812.13 (22.63)		763.72 (24.23)		556.79 (15.34)		607.62 (21.23)		762.60 (22.86)		742.18 (23.09)				
	Vigorous physical activity (minutes/7 days)		0		0		1174.72 (56.60)		1030.86 (75.38)		10.04 (9.96)		0.03 (0.07)				
	Total moderate-to-vigorous physical activity (minutes/7 days)		812.01 (22.58)		763.27 (24.21)		1732.28 (56.79)		1634.12 (68.09)		824.35 (23.98)		741.74 (23.18)				
	Average moderate-to-vigorous physical activity (minutes/day)		118.37 (2.78)		113.76 (3.27)		282.85 (7.58)		263.95 (10.74)		131.02 (3.17)		121.10 (3.17)				
**Sedentary behaviors**																	
	Average length of sedentary bouts (minutes/bout)		22.68 (0.16)		22.59 (0.15)		22.70 (0.19)		22.66 (0.19)		22.09 (0.23)		22.29 (0.21)				
	Daily average sedentary bouts (minutes/day)		328.01 (64.57)		316.64 (55.13)		319.40 (61.12)		314.95 (60.82)		311.77 (59.87)		317.42 (54.69)				
	Sedentary (minutes/7 days)		5659.73 (110.76)		5456.91 (119.63)		4629.65 (103.80)		4620.13 (95.25)		5032.84 (104.50)		5068.85 (91.27)				
**Dietary behaviors (questionnaire score)**																	
	Enjoyment of food		3.30 (0.07)		3.21 (0.06)		3.42 (0.08)		3.26 (0.07)		3.51 (0.08)		3.28 (0.07)				
	Emotional overeating		1.74 (0.05)		1.88 (0.06)		1.91 (0.06)		1.96 (0.06)		1.82 (0.06)		1.87 (0.06)				
	Satiety responsiveness		3.06 (0.06)		3.00 (0.06)		2.86 (0.06)		3.02 (0.05)		2.84 (0.06)		2.98 (0.07)				
	Slowness in eating		3.16 (0.08)		3.03 (0.07)		3.05 (0.08)		3.15 (0.07)		2.99 (0.08)		3.18 (0.07)				
	Desire to drink		2.33 (0.09)		2.38 (0.08)		2.30 (0.09)		2.32 (0.10)		2.36 (0.10)		2.29 (0.09)				
	Food fussiness		2.61 (0.07)		2.69 (0.06)		2.64 (0.07)		2.82 (0.07)		2.55 (0.07)		2.79 (0.06)				
	Emotional undereating		2.95 (0.14)		2.82 (0.07)		2.66 (0.08)		2.85 (0.07)		2.73 (0.07)		2.73 (0.08)				
	Food responsiveness		2.51 (0.05)		2.48 (0.06)		2.48 (0.07)		2.50 (0.07)		2.47 (0.07)		2.36 (0.06)				
**Sleep**																	
		Sleep problems (questionnaire score)	50.08 (0.66)		50.41 (0.61)		47.70 (0.72)		47.14 (0.68)		48.67 (0.65)		48.36 (0.70)				
		Sleep latency (minutes/day)	29.76 (1.83)		31.42 (1.78)		1.96 (0.31)		25.37 (0.89)		3.13 (0.49)		12.99 (1.69)				
		Sleep efficiency (%)	77.21 (0.64)		77.11 (0.71)		85.59 (3.06)		80.98 (0.66)		84.12 (0.59)		80.05 (0.64)				
		Sleep duration (minutes/day)	444.51 (3.77)		447.42 (4.02)		441.91 (6.92)		429.71 (5.18)		449.45 (5.55)		434.04 (5.96)				
**Screen time (** **minutes** **/day)**																	
		Weekdays	61.38 (5.28)		60.56 (4.47)		44.15 (2.47)		60.57 (4.42)		56.85 (5.40)		64.52 (5.46)				
		Weekends	117.14 (9.35)		130.50 (9.80)		56.42 (3.01)		130.31 (7.80)		107.28 (8.93)		134.85 (9.73)				

**Table 3:** Summary of generalized estimating equations (n=237).^a^

Variables						Within-group changes, adjusted mean difference (95% CI)						Group ⨯ time interaction effect	
						Posttest vs baseline	*P* value	Follow-up vs baseline	*P* value		*P* value		
**Light physical activity (minutes/7 days)**													.006
					Intervention	–1003.38 (–1150.10 to –856.66)	<.001	–214.82 (–349.85 to 79.78)	.10				
					Control	–745.95 (–936.82 to –555.09)	<.001	–107.03 (–251.27 to 37.20)	.44				
**MPA (** **minutes** **/7 days)**												.059	
				Intervention		–255.34 (–330.57 to –180.10)	<.001	–49.53 (–125.08 to 26.03)	.81				
				Control		–156.10 (–256.44 to –55.76)	<.001	–21.55 (–104.80 to 61.71)	.10				
**VPA (** **minutes** **/7 days)**												.02	
			Intervention			1174.72 (1008.57 to 1340.86)	<.001	10.04 (–19.20 to 39.27)	.10				
			Control			1030.86 (809.58 to 1252.13)	<.001	0.03 (–0.18 to –0.24)	.10				
**Total moderate-to-vigorous physical activity** **(** **minutes** **/7 days)**												.67	
		Intervention				920.26 (754.76 to 1085.77)	<.001	12.34 (–7.98 to 104.66)	>.99				
		Control				870.85 (673.25 to 1068.45)	<.001	–21.53 (–104.21 to 61.15)	>.99				
**Average moderate-to-vigorous physical activity (** **minutes** **/day)**												.36	
	Intervention					164.48 (142.50 to 186.45)	<.001	12.65 (–1.80 to 23.49)	.09				
	Control					150.19 (119.67 to 180.71)	<.001	7.34 (–3.33 to 18.01)	.65				
**Average length of sedentary bouts** **(** **minutes** **/bout)**												.68	
	Intervention					0.02 (–0.58 to 0.61)	.33	–0.59 (–1.30 to 0.12)	.22				
	Control					0.07 (–0.55 to 0.68)	>.99	-.302 (–0.98 to 0.37)	>.99				
**Daily average sedentary bouts (** **minutes** **/day)**												.27	
	Intervention					–8.70 (–32.85 to 15.44)	>.99	–16.41 (–40.37 to 7.54)	.66				
	Control					–1.19 (–24.80 to 22.42)	>.99	0.739 (–19.48 to 20.96)	>.99				
**Sedentary (** **minutes** **/7 days)**												.40	
	Intervention					–1030.08 (–1403.58 to –656.58)	<.001	–626.89 (–1026.67 to –227.11)	<.001				
	Control					–836.77 (–1207.94 to –465.61)	<.001	–388.06 (–770.28 to –5.84)	.04				
**Enjoyment of food (Questionnaire score)**												.38	
	Intervention					0.12 (0.10 to 0.34)	.049	0.21 (0.02 to 0.41)	.02				
	Control					0.07 (–0.17 to 0.30)	.43	0.07 (–0.14 to 0.29)	.48				
**Emotional overeating (Questionnaire score)**												.58	
	Intervention					0.17 (–0.04 to 0.37)	.25	0.08 (–1.00 to 0.26)	.09				
	Control					0.08 (–0.17 to 0.32)	>.99	–0.01 (–0.23 to 0.20)	>.99				
**Satiety responsiveness (Questionnaire score)**												.74	
	Intervention					–0.21 (–0.39 to –0.01)	.03	–0.21 (–0.40 to –0.03)	.007				
	Control					0.02 (–0.19 to 0.26)	.73	–0.01 (–0.20 to 0.17)	.38				
**Slowness in eating (Questionnaire score)**												.23	
	Intervention					–0.11 (–0.32 to 0.09)	.22	–0.18 (–0.39 to 0.04)	.22				
	Control					0.12 (–0.16 to 0.40)	>.99	0.14 (–0.07 to 0.36)	.76				
**Desire to drink (Questionnaire score)**												.61	
	Intervention					–0.02 (–0.30 to 0.24)	>.99	0.04 (–0.23 to 0.30)	>.99				
	Control					–0.06 (–0.38 to 0.26)	>.99	–0.09 (–0.38 to 0.19)	>.99				
**Food fussiness (Questionnaire score)**												.22	
	Intervention					0.03 (–0.18 to 0.24)	>.99	–0.07 (–0.28 to 0.14)	>.99				
	Control					0.13 (–0.10 to 0.37)	>.99	0.10 (–0.09 to 0.28)	>.99				
**Emotional undereating (Questionnaire score)**												.14	
	Intervention					–0.29 (–0.73 to 0.15)	.82	–0.22 (–0.65 to 0.20)	>.99				
	Control					0.03 (–0.23 to 0.28)	>.99	–0.09 (–0.35 to 0.17)	>.99				
**Food responsiveness (Questionnaire score)**												.50	
	Intervention					–0.02 (–0.24 to 0.19)	>.99	–0.03 (–0.23 to 0.16)	>.99				
	Control					0.02 (–0.21 to 0.26)	>.99	–0.02 (–0.32 to 0.09)	>.99				
**Sleep problems (score)**												.67	
	Intervention					–2.38 (–4.76 to –0.0006)	.049	–1.41 (–3.38 to 0.56)	.53				
	Control					–3.27 (–5.42 to –1.12)	<.001	–2.05 (–4.14 to 0.03)	.06				
**Sleep latency (** **minutes** **/day)**												.004	
	Intervention					–27.80 (–33.07 to –22.53)	<.001	–26.63 (–32.05 to –21.20)	<.001				
	Control					–6.05 (–31.22 to –20.89)	<.001	–18.43 (–25.65 to –11.20)	<.001				
**Sleep efficiency (%)^b^**												.002	
	Intervention					8.37 (0.54 to 5.73)	.006	6.89 (4.32 to 9.47)	<.001				
	Control					3.87 (1.47 to 6.27)	<.001	2.94 (0.79 to 5.08)	.001				
**Sleep duration (** **minutes** **/day)**												.12	
	Intervention					–2.61 (–26.20 to 20.98)	>.99	4.93 (–14.59 to 24.45)	>.99				
	Control					–17.71 (–36.07 to 0.64)	.069	–13.38 (–33.10 to 6.34)	.70				
**Screen time on weekdays (** **minutes** **/day)**												<.001	
	Intervention					–17.23 (–33.44 to –1.02)	.027	–4.52 (–20.82 to 11.78)	>.99				
	Control					0.01 (–15.34 to 15.36)	>.99	3.96 (–14.28 to 22.20)	>.99				
**Screen time on weekends (** **minutes** **/day)**												<.001	
	Intervention					–60.72 (–89.23 to –32.20)	<.001	–9.86 (–36.79 to 17.08)	.55				
	Control					–0.19 (–29.85 to 29.48)	>.99	4.36 (–29.72 to 38.44)	.78				

^a^Generalized estimating equations were conducted, adjusting for covariates, including preschoolers’ gender, age, height, weight, number of children with overweight or obesity, as well as parents’ age, height, weight, gender, educational level, income range, marital status, number of children in the household, and number of parents with overweight or obesity.

^b^Sleep efficiency refers to the percentage of time spent asleep while in bed. It is calculated by dividing total sleep time (in minutes) by total time in bed (in minutes). A sleep efficiency of 85% or higher is generally considered normal.

**Table 4:** Summary of post hoc analysis of generalized estimate equations whenever a group ⨯ time effect was detected (n=237).^a^

Variables	Posttest				Follow-up test		
	Adjusted mean difference (95% CI)	*P* value	Effect size (Cohen *d*)	Adjusted mean difference (95% CI)		*P* value	Effect size (Cohen *d*)
Light physical activity (minutes/7 days)	–151.61 (–418.18 to –96.67)	.002	0.28	–1.96 (–3.23 to 23.07)		.11	0.004
Vigorous physical activity (minutes/7 days)	138.47 (117.61 to 183.10)	.03	0.23	10.01 (–10.8 to 17.01)		.66	0.02
Sleep latency (minutes/day)	–21.04 (–16.07 to –6.00)	.005	0.78	–9.86 (–15.02 to –4.71)		<.001	0.55
Sleep efficiency (%)	4.61 (4.29 to 9.72)	<.001	0.34	4.07 (1.53 to 6.61)		<.001	0.30
Screen time on weekdays (minutes/day)	–16.42 (–30.83 to –2.01)	.01	0.25	–7.67 (–30.21 to 14.88)		.34	0.01
Screen time on weekend days (minutes/day)	–73.88 (–98.48 to –49.28)	<.001	0.46	–27.57 (–66.33 to 11.19)		.55	0.02

^a^Data for the intervention and control groups are presented. Bonferroni correction was applied to adjust for multiple pairwise comparisons. The analyses were further adjusted for covariates including preschoolers’ gender, age, height, weight, number of children with overweight or obesity, and parents’ age, height, weight, gender, educational level, income range, marital status, number of children in the household, and number of parents with overweight or obesity.

Compared with baseline, both the intervention and control groups showed significant within-group changes in LPA, MPA, VPA, total MVPA, and average MVPA at posttest. The trends indicated a significant decrease in time spent in LPA (intervention: −1003.38 minutes/week, 95% CI −1150.10 to −856.66, *P*<.001; control: −745.95 minutes/week, 95% CI −936.82 to −555.09, *P*<.001) and MPA (intervention: −255.34 minutes/week, 95% CI −330.57 to −180.10, *P*<.001; control: −156.10 minutes/week, 95% CI −256.44 to −55.76, *P*<.001), alongside an increase in MVPA (intervention: 164.48 minutes/day, 95% CI 142.50-186.45, *P*<.001; control: 150.19 minutes/day, 95% CI 119.67-180.71, *P*<.001) and VPA (intervention: 1174.72 minutes/week, 95% CI 1008.57-1340.86, *P*<.001; control: 1030.86 minutes/week, 95% CI 809.58-1252.13, *P*<.001).

Compared with the control group, preschoolers in the intervention group showed a significant reduction in time spent in LPA (adjusted mean difference −151.61 minutes/week, 95% CI −418.18 to −96.67, *P*=.002, Cohen *d*=0.28) and an increase in time spent in VPA (adjusted mean difference 138.47 minutes/week, 95% CI 117.61-183.10, *P*=.03, Cohen *d*=0.23) at posttest. At follow-up, there were no significant between-group differences in LPA (adjusted mean difference −1.96, 95% CI −3.23 to 23.07, *P*=.11) or VPA (adjusted mean difference 10.01, 95% CI −10.8 to 17.01, *P*=.66). No significant between-group differences were observed for other PA-related outcomes at either posttest or follow-up (MPA: *P*=.06; TMVPA: *P*=.67; and AMVPA: *P*=.36).

### Effectiveness of Parent-Based eHealth Intervention on Preschoolers’ Dietary Behaviors

No significant between-group differences in dietary behaviors were observed at either time point (enjoyment of food: *P*=.38; emotional overeating: *P*=.58; satiety responsiveness: *P*=.74; slowness in eating: *P*=.23; desire to drink: *P*=.61; food fussiness: *P*=.22; emotional undereating: *P*=.14; and food responsiveness: *P*=.50). Within-group analyses showed substantial changes in enjoyment of food and satiety responsiveness at posttest and follow-up in the intervention group, whereas no changes were observed in the control group. Specifically, the mean score for enjoyment of food in the intervention group increased from 3.30 (SD 0.07) at baseline to 3.42 (SD 0.08) at posttest (adjusted mean difference 0.12, 95% CI 0.10-0.34, *P*=.049) and further to 3.51 (SD 0.08) at follow-up (adjusted mean difference 0.21, 95% CI 0.02 to 0.41, *P*=.023). By contrast, the mean score for satiety responsiveness decreased from 3.06 (SD 0.06) at baseline to 2.86 (SD 0.06) at posttest (adjusted mean difference −0.21, 95% CI −0.39 to −0.01, *P*=.03) and to 2.84 (SD 0.06) at follow-up (adjusted mean difference −0.21, 95% CI −0.40 to −0.03, *P*=.007).

### Effectiveness of Parent-Based eHealth Intervention on Preschoolers’ Sleep

Significant within-group changes in preschoolers’ sleep-related outcomes were observed in both groups at posttest, except for sleep duration. Both the intervention and control groups showed a reduction in sleep problem scores at posttest (intervention: adjusted mean difference −2.38, 95% CI −4.76 to −0.0006, *P*=.049; control: adjusted mean difference −3.27, 95% CI −5.42 to −1.12, *P*<.001), with no significant between-group differences at either assessment (*P*=.67).

Although both the intervention and control groups showed a decreasing trend in sleep latency at posttest (intervention: adjusted mean difference −27.80 minutes/day, 95% CI −33.07 to −22.53, *P*<.001; control: −26.05 minutes/day, 95% CI −31.22 to −20.89, *P*<.001) and follow-up (intervention: −26.63 minutes/day, 95% CI −32.05 to −21.20, *P*<.001; control: −18.43 minutes/day, 95% CI −25.65 to −11.20, *P*<.001), the intervention group exhibited a significantly greater reduction than the control group at both posttest (adjusted mean difference −21.04 minutes/day, 95% CI −16.07 to −6.00, *P*=.005, Cohen *d*=0.78) and follow-up (adjusted mean difference −9.86 minutes/day, 95% CI −15.02 to −4.71, *P*<.001, Cohen *d*=0.55).

Both the intervention and control groups showed a significant increase in sleep efficiency at posttest (intervention: adjusted mean difference 8.37%, 95% CI 1.47-6.27, *P*=.006; control: 3.87%, 95% CI 1.47-6.27, *P*<.001) and follow-up (intervention: 6.89%, 95% CI 4.32-9.47, *P*<.001; control: 2.94%, 95% CI 0.79-5.08, *P*=.001). The improvement in sleep efficiency was more pronounced in the intervention group at both posttest (adjusted mean difference 4.61%, 95% CI 4.29-9.72, *P*<.001, Cohen *d*=0.34) and follow-up (adjusted mean difference 4.07%, 95% CI 1.53-6.61, *P*<.001, Cohen *d*=0.30) compared with the control group.

### Effectiveness of Parent-Based eHealth Intervention on Preschoolers’ Screen Time

Considering within-group changes, preschoolers in the intervention group showed a decline in screen time on both weekdays (adjusted mean difference –17.23 minutes, 95% CI –33.44 to –1.02, *P*=.027) and weekends (adjusted mean difference –60.72 minutes, 95% CI –89.23 to –32.20, *P*<.001). By contrast, preschoolers in the control group showed minimal changes in screen time on weekdays (adjusted mean difference –0.01 minutes, 95% CI –15.34 to 15.36, *P*>.99) and weekends (adjusted mean difference –0.19 minutes, 95% CI –29.85 to 29.48, *P*>.99). Although the intervention group maintained a reduced screen time at follow-up on weekdays (adjusted mean difference –4.52 minutes, 95% CI –20.82 to 11.78, *P*>.99) and weekends (adjusted mean difference –9.86 minutes, 95% CI –36.79 to 17.08, *P*=.55), these changes were not statistically significant compared with baseline.

Compared with the control group, preschoolers in the intervention group showed a decrease in screen time at posttest on weekdays (adjusted mean difference –16.42 minutes, 95% CI –30.83 to –2.01, *P*=.01, Cohen *d*=0.25) and weekends (adjusted mean difference –73.88 minutes, 95% CI –98.48 to –49.28, *P*<.001, Cohen *d*=0.46). At follow-up, the intervention group showed reductions in screen time on weekdays (adjusted mean difference –7.67 minutes, 95% CI –30.21 to 14.88, *P*=.34) and weekends (adjusted mean difference –27.57 minutes, 95% CI –66.33 to 11.19, *P*=.55) compared with the control group; however, these changes were not statistically significant.

## Discussion

### Principal Findings

This intervention successfully targeted parents to improve preschoolers’ PA, sleep, and screen time compared with the control group, with no significant effects on dietary behaviors. Regarding PA, preschoolers in the intervention group engaged in less LPA and more VPA at the 12-week posttest compared with the control group; however, these changes were not maintained at the 12-week follow-up. For sleep-related outcomes, the intervention group demonstrated reduced sleep latency and improved sleep efficiency at both the 12-week posttest and the 12-week follow-up compared with the control group. Additionally, preschoolers’ screen time in the intervention group significantly decreased on both weekdays and weekends at the posttest compared with the control group, but this reduction was not maintained at the 12-week follow-up.

### Effectiveness of Parent-Based eHealth on Preschoolers’ PA

Both groups exhibited changes in PA intensity from baseline to posttest, characterized by a decline in LPA and MPA, along with an increase in MVPA and VPA. These findings are consistent with previous studies [49-53], which suggested that LPA shows a decreasing trend from age 3 onward, while MVPA and VPA increase from age 3 to 5, plateau thereafter, and decline from age 7 to 9 [53]. Previous studies have explained that children explore their surroundings and develop self-awareness by demonstrating varying levels of competence in basic motor skills such as catching, rolling, running, and galloping [49,52]. Preschoolers often overestimate their motor competence, believing they are more skilled than they actually are; this inflated perception can motivate them to practice and master higher-level motor skills [54]. When children feel skilled, they are more likely to persist in improving their abilities [55]. Children with higher levels of actual motor competence and a greater repertoire of motor skills were more likely to engage in higher levels of PA, including increased MVPA and VPA [52]. Additionally, the “ActivityStat” hypothesis suggests that an imposed increase or decrease in PA in a single domain may lead to compensatory changes in another domain, helping to maintain stable overall PA and energy expenditure over time [56]. Accordingly, preschoolers in the intervention group maintained their total PA at a relatively constant level by adapting various mechanisms, such as altering the frequency, intensity, or duration of their PA [57]. Of particular note, this finding contrasts with the results reported by the systematic review of Zhou et al [25], which found no significant between-group differences in PA-related outcomes, as objectively measured by GT3X, among preschoolers following a parent-based online intervention. This discrepancy may be attributed to the greater number of PA-related modules in our study compared with the studies included in that review (n=4 vs n=1). It is plausible that the additional PA-related modules—including PA knowledge, group interaction, goal setting, tailored feedback, and weekly goal revision—captured more attention from parents in the intervention group, potentially motivating them to support their children in engaging in higher levels of PA.

Notably, parents in the intervention group were consistently educated on the recommendation that young children should engage in a minimum of 3 hours of PA/day, with a minimum of 1 hour consisting of MVPA. This targeted parental education and promotion of PA guidelines likely served as a key strategy driving the observed differences in LPA and VPA compared with the control group [58,59].

### Effectiveness of Parent-Based eHealth on Preschoolers’ Dietary Behaviors

From baseline to follow-up, preschoolers in the intervention group showed a significant increase in “enjoyment of food” (general interest in food) alongside a decline in “satiety responsiveness” (ability to stop eating in response to fullness cues). It is possible that the diet modules, regularly delivered to the intervention group, provided parents with a better understanding of balanced nutrition and healthy eating. This enhanced knowledge may have prompted parents to offer their children a wider variety of foods, contributing to increased food-approaching behaviors, including enjoyment of food [60].

In addition, many Chinese parents often misinterpret their children’s larger body weight and size as indicators of healthy growth, associating being overweight with affluence [61]. Parents may prefer their children to be chubbier and underestimate their status of overweight or obesity, making them more likely to pressure children to eat, an approach that has been associated with higher levels of enjoyment of food [62]. Chinese parental feeding practices are generally rooted in cultural values emphasizing not wasting food and ensuring children’s nutritional needs are met [63]. Parents often serve large portions of food as a way of expressing love and care for their children [60]. They tend to encourage their children to finish all the food on their plates during mealtimes, regardless of the child’s hunger and fullness cues [63]. Over time, these practices may reinforce the motivation to eat beyond physiological needs, contributing to higher enjoyment of food and lower satiety responsiveness in children [60].

Additionally, it is common in China for grandparents to co-reside with their adult children and serve as primary caregivers for their grandchildren [64]. Many grandparents, having lived through periods of food scarcity (such as the Great Famine of the 1950s), often associate increased food intake with successful caregiving and worry that their grandchildren may experience hunger [65]. As a result, grandparents may encourage excessive eating to prevent malnutrition, pressuring their grandchildren to eat even when not hungry, which can increase children’s enjoyment of food while reducing their responsiveness to satiety cues [65].

In previous parent-based eHealth intervention studies, the focus was primarily on preschoolers’ dietary consumption (such as noncore foods, fruits and vegetables, and sugar intake) rather than on eating behaviors [25]. Consequently, there is limited scope for direct comparison. The allocation of preschoolers in our study resulted in an unequal distribution of eating behaviors between the intervention group (eg, enjoyment of food, 47/120, 39.2%; satiety responsiveness 14/120, 11.7%) and the control group (eg, enjoyment of food, 37/117, 31.6%; satiety responsiveness 13/117, 11.1%) at baseline. Such uneven allocation may have influenced the overall intervention effects on preschoolers’ dietary behaviors. Future studies with a more balanced distribution of eating behaviors are therefore warranted.

Additionally, eating behaviors, which develop during early childhood and reflect individual appetitive traits associated with the regulation of food intake, have been found to be influenced by parenting practices and styles. For instance, feeding practices that involve encouragement and rewards can enhance food-approaching behaviors (such as enjoyment of food) and may even reduce satiety responsiveness in preschoolers [66]. High parental self-efficacy in feeding has also been linked to healthier eating behaviors in preschoolers [67]. In addition, an authoritarian parenting style has been associated with increased food responsiveness, emotional overeating, enjoyment of food, and satiety responsiveness [68]. Future studies exploring the mediating effects of parenting practices and styles are warranted, as mediation analysis can provide a better understanding of the factors that influence the effectiveness of parent-based eHealth interventions on preschoolers’ eating behaviors.

### Effectiveness of Parent-Based eHealth on Preschoolers’ Sleep

Improvements in sleep problems, sleep latency, and sleep efficiency among preschoolers in the intervention group were observed at posttest following the parent-based eHealth intervention, with gains in sleep latency and sleep efficiency sustained at follow-up. These enhancements may be explained by the role of PA in promoting several sleep variables, including sleep-onset latency, sleep efficiency, and total sleep duration [69-71]. According to energy conservation and body restoration theories, sleep duration and the amount of slow-wave sleep increase as a function of higher energy expenditure [72]. Specifically, PA depletes energy reserves, particularly glycogen stores in muscles and the liver. As energy expenditure increases and these stores are reduced, the body signals a need for recovery, enhancing the drive for sleep. This can lead to longer sleep durations and a greater proportion of slow-wave sleep, which is particularly crucial for physical recovery and is where many restorative processes occur [72].

Previous studies further reported that PA triggers the release of neurotransmitters such as serotonin and norepinephrine, which are important for mood regulation and relaxation, potentially contributing to improved sleep onset and maintenance [73]. When daytime MVPA was increased by 10%, sleep latency decreased by 12.15 minutes/day [74]. Higher levels of MVPA were also significantly associated with better sleep efficiency and reduced sleep fragmentation [70]. Two studies showed that MVPA improved sleep quality and alleviated sleep problems by decreasing sleep latency, increasing total sleep time, and reducing presleep anxiety [75,76]. Preschoolers in the intervention group exhibited a notable increase in total MVPA, from approximately 812 minutes at baseline to 1732 minutes at posttest, and then 824 minutes at follow-up, which may have contributed to the observed improvements in sleep problems, sleep latency, and sleep efficiency.

Beyond the biological perspective, parents in the intervention group were regularly provided with educational knowledge related to sleep, including adequate sleep and optimal sleep environments. This enhanced knowledge likely helped parents better understand the factors contributing to good sleep hygiene and the importance of maintaining consistent bedtimes. Regular bedtime routines improve sleep quality—such as reducing sleep latency and night wakings in young children—and help synchronize the internal biological clock, promoting self-regulation of the sleep-wake cycle and facilitating the natural sleep process [77,78]. Children with shorter sleep latency are able to spend a greater proportion of time in actual bed sleep and are less likely to experience nighttime disruptions and awakenings, which reduces interruptions and enhances sleep efficiency [79].

The insignificant difference between the 2 groups in sleep problems may be attributed to 2 factors. First, the high proportion of preschoolers with severe sleep problems (ie, total scores above 41) at baseline in both the intervention (109/120, 90.8%) and control groups (107/117, 91.5%) suggests that the improvements observed in parent-reported sleep problems at posttest may have been influenced by a regression-to-the-mean effect, rather than solely by the impact of the intervention [80]. Individuals initially identified with high values on the outcome measure are likely to show lower values upon remeasurement, even in the absence of any intervention [80]. Thus, it is possible that their sleep issues may have naturally improved over time, regardless of whether they received an intervention. Additionally, unlike sleep latency and efficiency, which were objectively assessed, preschoolers’ sleep problems were evaluated using parent-reported measures. This introduces the potential for parents to intentionally or unintentionally misreport information [81].

### Effectiveness of Parent-Based eHealth on Preschoolers’ Screen Time

The intervention group showed a significant reduction in screen time on both weekdays and weekends compared with the control group. This finding is consistent with previous studies [82-84]. Parent-child attachment, defined as a mutually satisfying relationship, may influence screen time, as lower attachment levels have been associated with increased screen time due to reduced parental monitoring [82]. Stronger father-child and mother-child attachment was correlated with decreased screen time, with ego resilience mediating this relationship [83]. Meaningful interactions, such as engaging in enjoyable activities together, can strengthen parent-child attachment [84]. In the intervention group, parents were encouraged to participate in PA with their children, fostering bonding and positive memories, which likely contributed to the reduction in screen time.

### Strengths and Limitations

This study addressed weaknesses identified in previous research, such as balancing the intervention content and minimizing the impact of the sequence in which the content was delivered on the outcomes. Integrating the entire intervention process with 3 critical components and 4 processing steps based on social cognitive theory provided a consolidated rationale for the intervention design. Objectively assessed data on preschoolers’ PA and sleep enhanced the accuracy and reliability of the results.

The findings of this study should be interpreted in light of certain limitations. First, measurements of preschoolers’ dietary behaviors and sleep problems relied on subjective questionnaires, which involved self-reported data. Consequently, parents may have misreported this information, either intentionally or unintentionally, potentially affecting the results in both groups. Second, because the posttest coincided with the Chinese Lunar New Year holiday, many participants were unable to return to school to complete the measurements within the proposed time frame, resulting in a high attrition rate (181/237, 76.4%). Third, a greater-than-expected proportion of children were classified as having an active lifestyle (engaging in 180 minutes of PA daily, with MVPA meeting 60 minutes), which may have diluted the intervention’s effect on MVPA. Finally, as participants were mainly from underdeveloped regions in China, the results may not generalize to preschoolers in developed regions.

### Practical Implications

Chinese parents often feel a strong sense of responsibility to guide and correct their children’s behavior, which can result in a high level of controlling behavior from an early age [85]. Interventions aimed at improving the lifestyle behaviors of Chinese preschoolers should not only target the children’s behavior but also give greater attention to parenting practices and styles. Future research is essential to examine how these parenting approaches mediate the effectiveness of parent-based eHealth interventions on preschoolers’ PA, diet, and sleep. Moreover, this intervention has potential for scalability and broad reach. Studies with larger sample sizes are needed to translate this effective parent-based eHealth intervention to other age groups and regions of China.

### Conclusions

Within the Chinese cultural context, educating parents about healthy lifestyles via social media effectively improved preschoolers’ VPA, sleep latency and efficiency, and screen time compared with the control group. The insights gained from this study may help address the unhealthy lifestyle behaviors commonly observed in contemporary young children. Considerably more work is needed to leverage cutting-edge technological advancements to help families across broader regions create healthy living environments for their children. Such efforts can not only enhance individual health and family well-being but also reduce the societal financial burden associated with treating diseases resulting from poor lifestyle habits.

## Appendix

Multimedia Appendix 1 CONSORT-eHEALTH checklist (V 1.6.1).

## Abbreviations

CONSORT: Consolidated Standards of Reporting Trials
CPM: counts per minute
LPA: light physical activity
MPA: moderate physical activity
MVPA: moderate-to-vigorous physical activity
SMART: Specific, Measurable, Achievable, Relevant, and Time-bound
VPA: vigorous physical activity

## Data Availability

The data supporting the findings of this study are available from the corresponding author (PWCL) upon reasonable request.
